# Real-World Experience With Proactive Therapeutic Drug Monitoring During Infliximab Reintroduction

**DOI:** 10.1093/crocol/otab048

**Published:** 2021-07-13

**Authors:** Inessa Normatov, Daniela Fluxa, Jingzhou D Wang, Jacob E Ollech, George E Gulotta, Shivani Patel, Maria A Quintero, Bety De la Torre, Norma Solis, Oriana M Damas, Amar R Deshpande, David H Kerman, Maria T Abreu, David T Rubin

**Affiliations:** 1 University of Chicago Medicine Inflammatory Bowel Disease Center, Chicago, Illinois, USA; 2 Crohn’s and Colitis Center, University of Miami Miller School of Medicine, Miami, Florida, USA

**Keywords:** infliximab, drug holiday, infliximab restart, infusion reaction, therapeutic drug monitoring

## Abstract

**Background:**

Interruptions in infliximab therapy are associated with the development of antibodies to infliximab (ATI), infusion reactions (IRs), and loss of response. Despite these challenges, recent observational studies suggest that reinitiating infliximab after a drug holiday can be safe and effective. We assessed the utility of our protocol for restarting infliximab using early serum infliximab and ATI measurements.

**Methods:**

A retrospective cohort study of patients restarted on infliximab after at least a 6-month drug holiday. The cohort was divided into 2 groups: a “therapeutic drug monitoring (TDM) group,” those who had serum infliximab and ATI measured 1–3 weeks after first reinduction dose, and a “non-TDM group.” Outcomes included results of TDM, occurrence of immediate IR (IIR) and delayed hypersensitivity reactions, and medication persistence at 14 weeks and 1 year.

**Results:**

About 76 patients were included: 49 in the TDM group and 27 in the non-TDM group. Of 76, 67 (88%) patients tolerated the first reinduction dose without IR. Formation of ATI was seen in 17 of 49 (35%) patients and was associated with longer drug holidays. Most did not experience IR during the entire therapy course—in 26 of 32 (81%) without ATI and 20 of 27 (74%) in the non-TDM group. Infliximab persistence at 14 weeks and 1 year was 76% and 57% for the cohort, respectively.

**Conclusion:**

Infliximab can be safely and effectively restarted after a drug holiday. We suggest performing TDM with a drug-tolerant assay 1–3 weeks after the first reinduction infusion as a means to identify patients at risk for severe IIR at the second dose.

## Introduction

The recognition of tumor necrosis factor (TNF) as a key proinflammatory cytokine in Crohn’s disease (CD) and ulcerative colitis (UC) and the subsequent development of biologic drugs targeting TNF have redefined the management of inflammatory bowel disease (IBD). Infliximab (IFX), a chimeric IgG1 monoclonal antibody against TNF, is highly effective in the treatment of luminal and fistulizing CD as well as UC.^1–4^ Despite this, a significant proportion of patients will have an unsatisfactory primary response to IFX induction or will experience loss of response (LOR) over time following an initial response.^5–7^ The annual risk for LOR to IFX is approximately 13% per patient-year of treatment.^8^

Among patients with LOR to IFX, one important mechanism is immunogenicity, that is, host antibodies against IFX.^9^ Multiple studies have demonstrated an association between the presence of antibodies to IFX (ATI) and LOR, as well as infusion reactions (IRs).^10, 11^ The HLA-DQA1*05 allele has been associated with increased immunogenicity to IFX and adalimumab.^12^ Other risk factors for development of ATI include episodic exposure to IFX compared to scheduled maintenance therapy.^2, 10^ Long drug holidays have been associated with a high risk for immediate IR (IIR) and delayed hypersensitivity reactions (DHRs).^13, 14^ However, recent studies evaluating IFX restart following a drug holiday have suggested that it may be performed safely and effectively, even for patients who previously discontinued IFX therapy due to LOR.^15–18^

We sought to clarify the role of proactive assessment of therapeutic drug levels and ATI in the setting of restarting IFX therapy in patients who were previously exposed but who had a treatment interruption. We hypothesized that screening for ATI after the first reinduction dose could be used to identify those patients at risk for a subsequent hypersensitivity reaction to IFX. Our study proposes an algorithm for safely reinitiating IFX in select patients.

## Methods

### Study Design and Patients

**Figure 1.**
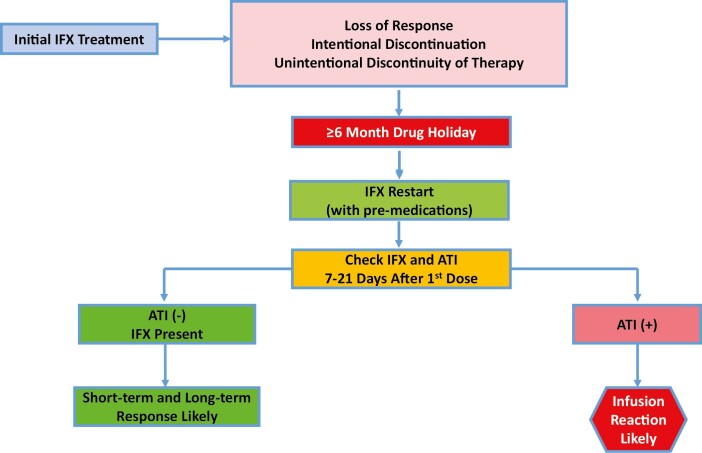
Algorithm for restarting infliximab.

This was a retrospective cohort study performed at 2 IBD centers in the United States: The University of Chicago Medicine Inflammatory Bowel Disease Center and The Crohn’s and Colitis Center at the University of Miami Miller School of Medicine. Through a systematic review of our medical records from January 2014 onward, we identified all patients with IBD who had previously received IFX treatment, had a drug interruption/holiday of minimum 6 months, and who were subsequently restarted on IFX. At the treating clinician’s discretion, some patients followed the outlined protocol (Figure 1) and had proactive therapeutic drug monitoring (TDM) performed following the first reinduction dose, while others continued with reinduction doses without TDM or with TDM prior to the first reinduction dose. Based on this, the cohort was divided into 2 groups: a “TDM group,” which included patients who had serum IFX levels and ATI measured (as permitted based on the assay used) 1–3 weeks after the first reinduction dose, and a “non-TDM group.” For the patients who had TDM and were found to have ATI, subsequent infusions were usually held.

The patients were premedicated at the time of infusions. All patients received intravenous diphenhydramine and oral acetaminophen. Most patients also received intravenous corticosteroids. Patients received their second infusion dose between 2 and 4 weeks from the first infusion. All but 2 patients had a minimum follow-up period of 1 year at the time of the last data analysis.

For this analysis, we used the World Health Organization nomenclature for the classification of IRs to immunoglobulins into 2 types—immediate and delayed.^19^ IIRs are defined as those during the infusion or within 1–2 hours of its completion and DHRs first manifest more than 24 hours post-infusion, usually producing serum sickness-type symptoms. The reactions were further determined to be mild, moderate, or severe. Mild and moderate reactions subside after pausing the infusion and allow for the completion of the infusion after a break, with or without intravenous corticosteroids, or at a slower infusion rate. Severe reactions are those which necessitate cessation of therapy due to significant cardiovascular or respiratory symptoms.

### Therapeutic Drug Assays

Analysis of serum samples for TDM was performed by several different laboratories. At The University of Chicago Medicine Inflammatory Bowel Disease Center, most samples were analyzed using solid-phase detection techniques (electrochemiluminescence immunoassay and enzyme-linked immunosorbent assay) performed by LabCorp, Mayo Clinic Laboratories, Quest Diagnostics, and Inform Diagnostics. About half of the patients had ATI measured as a “reflex” only if the IFX level was under a predefined threshold. At The Crohn’s and Colitis Center at the University of Miami Miller School of Medicine, all but one patient had TDM performed using the PROMETHEUS Anser IFX homogeneous mobility shift assay. Regardless of the assay used, any level of detectable antibodies was considered ATI positive.

### Data Collection

The medical records were reviewed retrospectively to obtain standard demographic data, smoking status, IBD clinical characteristics (IBD type, age at IBD diagnosis, disease extent/location, and Crohn’s phenotype), reason for initial discontinuation of IFX treatment (LOR/incomplete response, remission, pregnancy, medication adverse effect, loss to follow-up/insurance-related, surgery, or patient preference), duration of drug holiday, and other IBD therapies used prior to restarting IFX therapy.

We also collected the following information on the IFX restart period: age at restart, therapy used (IFX vs the biosimilar IFX-dyyb), concomitant immunomodulator (IMM; thiopurine vs methotrexate), reason for stopping therapy (incomplete response, high ATI presence, IIR and/or DHR, other medication adverse effect, patient preference, or loss to follow-up), duration on therapy, and characteristics of any IIR or DHR. Additionally, for the TDM group, serum IFX level, ATI level, and the laboratory performing the testing were recorded.

The co-primary endpoints were persistence of IFX therapy, noted at 14 weeks and 1 year, and safety of restarting the therapy. We used persistence of therapy as an indicator for response to therapy and we defined safety as the absence of IIR.

### Statistical Analysis

Statistical analyses were performed using the Statistical Analysis Systems (SAS) Software program (version 9.4; SAS Institute Inc.). The α-error was set at 0.05 and reported *P* values are 2-sided. Descriptive statistics were reported as percentages, mean values, and standard errors of the mean. Univariate analysis was performed to compare baseline characteristics between patients under TDM and patients without TDM by using chi-square tests for categorical variables and analysis of variance (ANOVA) for continuous variables. Logistic regression analysis was used to assess the relationship between the class of TDM and clinical outcome of interests, which include initial IR, subsequent IR, DHR, ATI formation, 14-week medication persistence, and 1-year medication persistence. ANOVA was used to compare the length of drug holidays and 1-year medication persistence. A Kaplan–Meier curve was used to compare the cumulative probability of medication persistence between ATI-negative TDM and non-TDM groups.

## Results

### Patient Characteristics

A total of 76 patients were included, 49 patients in the TDM group and 27 in the non-TDM group. Patient baseline characteristics are given in Table 1. The 2 groups were similar in disease characteristics, smoking status, reason for discontinuation of the first IFX course, and duration of drug holiday. The groups differed in the rate of concomitant IMM use at the IFX restart course, with the non-TDM group having double the patients on IMM compared with the TDM group, as well as history of exposure to other medications prior to restart, particularly exposure to vedolizumab, ustekinumab, and thiopurines.

**Table 1.** Baseline patient characteristics

	TDM group (*n* = 49)	Non-TDM group (*n* = 27)	*P*
Age at IBD diagnosis, mean	24.10 ± 1.89	20.22 ± 2.55	.23
Age at restart, mean	36.53 ± 2.13	30.41 ± 2.86	.09
Gender (female/male)	29/20	10/17	.06
IBD type, *n* (%)			.42
CD	38 (78%)	24 (89%)	
UC	10 (20%)	3 (11%)	
IBD-U	1 (2%)	0 (0%)	
Crohn’s disease behavior Montreal classification, *n* (%)			.38
B1	14 (37%)	13 (54%)	
B2	7 (18%)	4 (17%)	
B3	17 (45%)	7 (29%)	
Perianal involvement	15 (39%)	8 (33%)	
Crohn’s disease location Montreal classification			
L1	5 (13%)	2 (8%)	.52
L2	8 (21%)	8 (33%)	
L3	25 (66%)	14 (58%)	
+L4	1 (3%)	4 (17%)	
UC extension			.57
E1	0	0	
E2	2	1	
E3	9	2	
Smoking status			.15
Never	36	20	
Past	11	3	
Current	2	4	
Concomitant IMM at restart			.0029
Yes, n (%)	17 (35%)	19 (70%)	
Methotrexate/thiopurine	7/10	8/11	
Drug holiday (months)	49.81 ± 6.10	42.81 ± 8.22	.50
Reason for discontinuation of first IFX course			
Loss of response or partial response	23 (47%)	11 (41%)	.24
Remission	6	1	
Pregnancy	1	0	
Adverse effect	1	3	
Loss to follow-up or insurance-related	6	8	
Surgery	6	2	
Convenience or patient preference	5	1	
Unknown	1	1	
Prior medications			
Other anti-TNF	24 (49%)	14 (52%)	.81
Vedolizumab	19 (39%)	4 (15%)	.03
Ustekinumab	10 (20%)	1 (4%)	.048
Thiopurine	27 (55%)	22 (81%)	.02
Methotrexate	11 (22%)	9 (33%)	.30
Thalidomide	1 (2%)	1 (4%)	.66
Budesonide	10 (20%)	5 (19%)	.84
5-ASA	22 (45%)	17 (63%)	.13
Calcineurin inhibitor	8 (16%)	3 (11%)	.54
Tofacitinib	1 (2%)	0 (0%)	.46
Natalizumab	1 (2%)	0 (0%)	.45

Abbreviations: 5-ASA, 5-aminosalicylic acid; CD, Crohn’s disease; IBD, inflammatory bowel disease; IFX, infliximab; IMM, immunomodulator; TDM, therapeutic drug monitoring; TNF, tumor necrosis factor; UC, ulcerative colitis.

The reason for discontinuation of the first therapeutic course with IFX was LOR or insufficient response to IFX in nearly half (45%) of our cohort. The specifics regarding the nature of the first therapy course, such as the schedule of administration (episodic vs continuous), whether dose optimization was attempted and whether evaluation for disease activity and presence of ATI prior to discontinuation were assessed, were not available to us due to the retrospective nature of this study. Importantly, patients who had reported previous IRs to IFX were not offered an attempt at IFX reinitiation.

### IIRs With First Restart Infusion

Our protocol measured ATI after the first reinduction dose which risks missing patients who have preexisting ATI. The cohort had a total of 3 patients (4%) who experienced an IIR during the first restart dose: 2 were in the TDM group and 1 in the non-TDM group. Both patients in the TDM group were found to have ATI (100 U mL^−1^ through Prometheus and 64 ng mL^−1^ through LabCorp) following the first restart infusion. In these 3 patients, the IIRs were non-life-threatening and they were able to complete the first dose after a pause in the infusion and additional diphenhydramine. The specific reactions were jaw pain in the patient with ATI of 100 U mL^−1^ (Prometheus), pruritis and throat discomfort during the infusion in the patient with ATI of 64 ng mL^−1^ (LabCorp), and sensation of body warmth in the non-TDM group patient.

**Figure 2.**
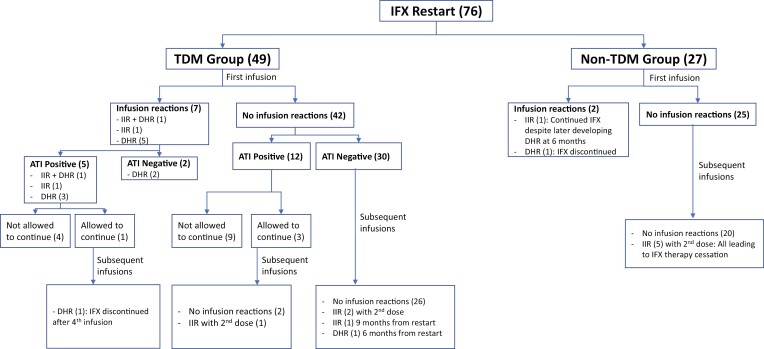
Infusion reactions.

One had discontinuation of therapy following the first infusion (patient with ATI of 100 U mL^−1^ through Prometheus) and the others were continued with further infusions. The incidence of IIR with the first dose was similar between the 2 groups (*P* = .94). IRs are summarized in Figure 2.

### DHRs With First Restart Infusion

Our cohort identified 7 patients (9%) who experienced a DHR with the first restart dose—6 were in the TDM group, 4 with positive ATI. One of these patients also experienced an IIR with the first dose (ATI of 64 ng mL^−1^ through LabCorp). The other 3 had ATI of 907 U mL^−1^ (Mayo), 309 µg mL^−1^ (Arup), and 24.5 U mL^−1^ (Prometheus). The patient in the non-TDM group developed a rash and hypotension more than 24 hours after the infusion for which he was admitted to another hospital. Unfortunately, the patient was lost to follow-up after this event and no further information could be obtained.

### IRs With Subsequent Infusions

#### TDM group

Of the 49 patients who were restarted on IFX with TDM at our centers, 17 were found to have ATI in serum samples drawn 1–3 weeks following the first restart infusion. Of these patients, 13 were not allowed to continue with further IFX therapy as per the outlined protocol (Figure 1).

Four of the patients with ATI continued with further therapy at the discretion of the treating physician, with the rationale being that ATI levels were considered low and/or due to the necessity of the therapy. The levels of ATI in these 4 patients were 220 ng mL^−1^ (LabCorp), 208 ng mL^−1^ (LabCorp), 64 ng mL^−1^ (LabCorp), and 8.8 U mL^−1^ (Prometheus). The patient with an ATI level of 220 ng mL^−1^ (LabCorp) was admitted for the second dose so that it could be given in a more controlled setting and she could be observed thereafter. Two of the 4 (those with ATI levels of 220 ng mL^−1^ through LabCorp and 8.8 U mL^−1^ through Prometheus) responded well to therapy, without any immune-related adverse events and were still receiving IFX at their last follow-up (at 27 months and 19 months from restart, respectively). The patient with an ATI level of 208 ng mL^−1^ (LabCorp) developed an IIR with the second restart dose (flushing and tachycardia). He was able to complete the infusion with additional medications and a pause but further therapy was discontinued. The patient with an ATI level of 64 ng mL^−1^ (LabCorp) who had throat discomfort and pruritis with the first infusion developed bothersome classic serum sickness-like DHR prompting discontinuation of IFX after 4 doses. This was surprising as this level of ATI is generally considered low and thus expected to be clinically insignificant.

The remaining 32 patients did not have detectable ATI (for non-Prometheus tests, ATI could not be measured if serum IFX level was not under a set threshold); these patients were allowed to continue with the treatment course. Three patients without ATI developed subsequent IIR—2 during the second infusion and 1 after a maintenance infusion 9 months from restart (developed urticaria). All 3 IIRs were mild to moderate in severity. The patients were able to complete the infusion following a pause, with restart at a slower rate and an additional dose of intravenous diphenhydramine; however, IFX therapy was discontinued thereafter. Their serum IFX levels following the first restart dose were 2.09 mcg mL^−1^ (Quest), 13 mcg mL^−1^ (Mayo), and 19 mcg mL^−1^ (Mayo). The patient with serum IFX of 13 mcg mL^−1^ (Mayo) had TDM following the IIR (pruritis and urticaria) with the second infusion and was found to have a serum IFX level of 1.3 μg mL^−1^ with ATI of 705 U mL^−1^ (Prometheus). The patient who had an IIR at 9 months from restart (TDM after the first dose: IFX 19 mcg mL^−1^ through Mayo, did not reflex to ATI testing) continued therapy until a year from restart. She has Crohn’s disease and had a good response to re-therapy, with normalization of C-reactive protein and calprotectin. Interestingly, her therapy was discontinued because she was found to have high ATI with no IFX level on TDM at 1 year from restart (Prometheus lab: IFX <1 μg mL^−1^, ATI 70.4 U mL^−1^) and was transitioned to adalimumab.

One patient had no detectable serum IFX but also no ATI at first TDM (performed at Mayo). Subsequent TDM tests, done through various laboratories (Mayo, Miraca, Inform Diagnostics, and Quest), showed detectable IFX levels without ATI. She required dose intensification over the course of therapy, most recently receiving 10 mg kg^−1^ every 6 weeks, with clinical response to therapy, without any immune-related adverse events, and was still receiving IFX at her last follow-up (67 months from restart).

In addition to the 6 patients in the TDM group who developed DHR with the first dose, another patient without ATI developed a DHR 6 months from restart.

#### Non-TDM group

Five patients in the non-TDM group developed moderate-to-severe IIR; 4 of these patients required immediate termination of the infusion and treatment with intravenous steroids and diphenhydramine. In all cases, this was during the second restart infusion and further IFX therapy was discontinued. Two of these patients had serum IFX and ATI measured prior to the second dose; however, the results were not available at the time of the second infusion. They both had high levels of ATI—47.7 U mL^−1^ and 100.0 U mL^−1^ (both through Prometheus).

#### Comparison of primary outcomes

When comparing rates of IR between the ATI-negative TDM group and the non-TDM group, there was no statistical difference in rates of IIR (*P* = .31) or DHR (*P* = .79). However, IRs were more severe in the non-TDM group.

When evaluating IFX persistence for the patients in the TDM group who did not develop ATI following the first infusion, 28 of the 32 (88%) remained on IFX at 14 weeks and 18 of 30 (60%) remained at 1 year from restart. Two patients were excluded from the 1-year persistence analysis as they did not reach the 1-year mark at the time of analysis.

**Figure 3.**
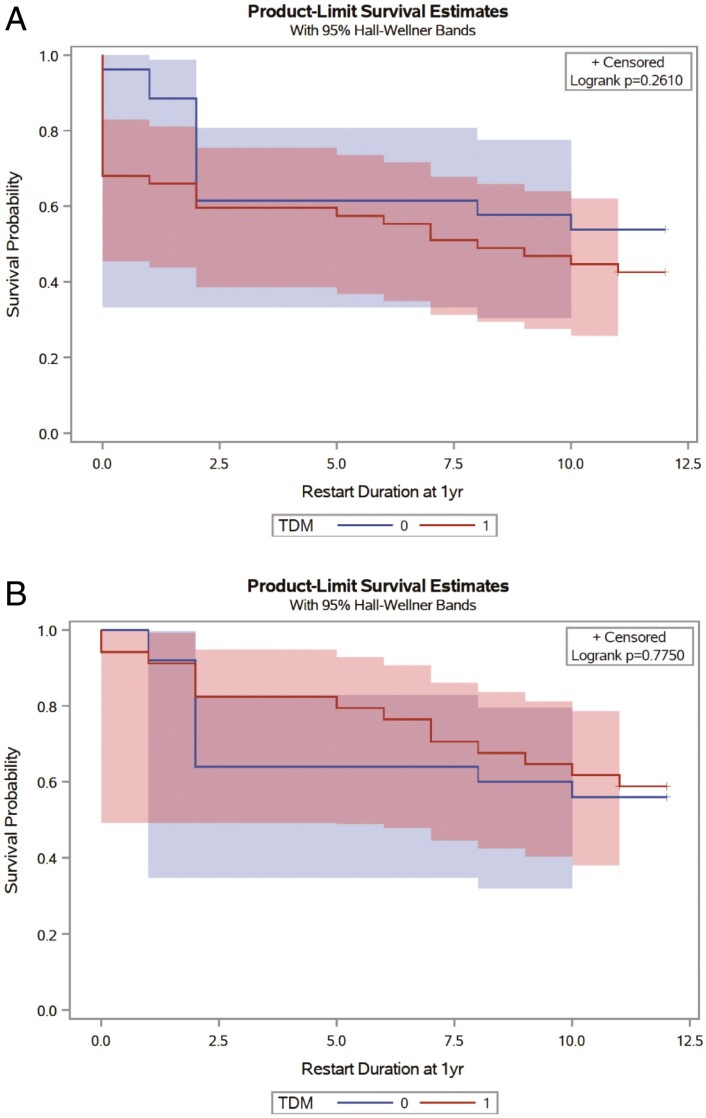
KM curve analysis for IFX persistence. A, KM curve analysis comparing IFX persistence in the TDM group vs non-TDM group. Blue = non-TDM group; Red = TDM group (ATI negative and ATI positive). B, KM curve analysis comparing IFX persistence in ATI-negative TDM group vs non-TDM group. Blue = non-TDM group; Red = ATI-negative TDM group. ATI, antibodies to infliximab; IFX, infliximab; KM, Kaplan–Meier; TDM, therapeutic drug monitoring.

Of 27 patients in the non-TDM group, 18 (67%) were on IFX at 14 weeks and 14 (54%) had IFX persistence at 1 year. One patient did not reach the 1-year follow-up mark from restart. The rate of medication persistence at either time point was not statistically different from that of the ATI-negative patients in the TDM group (*P* = .64 for 14 weeks and .35 for 1 year). Results are summarized in Table 2. KM curve analysis for IFX persistence is shown in Figure 3.

**Table 2.** Comparison of clinical outcomes

	ATI-negative TDM group	Non-TDM group	*P*
Immediate infusion reactions (does not include the first dose)	3/32 (9.4%): 2 during the second infusion, 1 at 9 months from restart	5/27 (18.5%): all during the second infusion and lead to treatment cessation	.31
Delayed hypersensitivity reactions	3/32 (9.4%)	2/27 (7.4%)	.79
14-week medication persistence	28/32 (87.5%)	18/27 (66.7%)	.054
1-year medication persistence	18/30 (60%)^a^	14/26 (53.8%)^b^	.64

Abbreviations: ATI, antibodies to infliximab; TDM, therapeutic drug monitoring.

^a^No 1-year data for 2 patients.

^b^No 1-year data for 1 patient.

#### Predictors of outcomes

We next looked at the relationship between clinical parameters and IR. Univariate logistic regression analysis showed that IR rates were not associated with age at IBD diagnosis (*P* = .83), age at IFX restart (*P* = .76), smoking status (*P* = .72), duration of drug holiday (*P* = .35), or the use of concomitant IMM at restart (*P* = .49).

In the TDM group, the presence of ATI 1–3 weeks following the first restart dose was associated with a longer drug holiday (67.6 months vs 41.5 months, *P* = .0385). There was no association with prior concomitant IMM use (OR 0.53, CI: 0.17–1.6).

Logistic regression analysis was performed by including patients with negative ATI in the TDM group and all non-TDM patients. Patients with positive ATI were not included as the protocol intended to discontinue IFX after ATI was detected.

IFX persistence at 14 weeks was associated with a younger age at IBD diagnosis (20.3 vs 26.9 years, *P* = .0360), younger age at restart of IFX (31.3 vs 39.6 years, *P* = .0193), a shorter drug holiday (35.3 vs 68.0 months, *P* = .0009), and the concomitant use of an IMM at restart (OR 3.5, CI: 1.3–9.5).

IFX persistence at 1 year was associated with a younger age at restart of IFX (29.6 vs 38.8 years, *P* = .0092) and a shorter drug holiday (28.6 vs 66.3 months, *P* < .0001). There was no association with concomitant IMM use (OR 1.3, CI: 0.5–3.9) or the reason for discontinuation of the first course of IFX therapy.

## Discussion

We examined the use of TDM in a consecutive cohort of patients with IBD who were being retreated with IFX following a drug holiday to identify patients who are at risk of serious IR and unlikely to benefit from IFX retreatment. Patients with IBD who develop ATI have double the risk of developing any IIR and nearly a 6-fold risk of serious IRs,^20^ and it is known that an interruption in drug therapy increases the risk of IR^14, 21^ as well as the development of ATI.^11, 22^ Baert et al studied patients restarted on IFX after a drug holiday (median of 15 months) and retrospectively analyzed their bio-banked serum samples for IFX trough levels and ATI. They demonstrated that measuring ATI prior to the first restart dose was not helpful in predicting response or safety of reinitiation as all patients had undetectable ATI. However, the absence of ATI in an early sample after reexposure to IFX was associated with both short-term responses and decreased risk of IR.^18^ Ours is the first study to examine this proactively and in real-life practice.

We show that assessing serum concentrations of drug and antidrug antibody status after the first restart infusion can identify patients who develop ATI and are thus at risk for early IIR as well as serious IIR. The logic of such a protocol is that after a long dose interruption, memory B cells that recognize IFX have not been stimulated by cognate antigen and thus the ATI measurement will be negative if done prior to the first restart dose. Yet once a patient with memory B cells specific for IFX is rechallenged with antigen, they then generate ATI. We therefore reasoned that measurement of ATI after the first restart dose would be better than before restart.

Previous studies on reinitiation of IFX therapy have reported IR in 0%–39% of patients.^14–18, 23, 24^ Findings of these previous studies are summarized in Table 3. In our cohort, 4% of the patients had an IIR with the first infusion and 12% with subsequent infusions. Using the TDM strategy, we identified 17 of 49 patients who developed ATI after the first dose; 13 of whom did not get a second infusion. This strategy missed 2 patients who developed IIR with the first dose, 6 patients who developed a DHR after the first dose, and 2 patients who developed an IIR with the second dose in spite of negative ATI. These latter patients had IFX levels checked using an assay that only reflexes to checking ATI if levels of the drug are undetectable. It is possible that even these could have been avoided with a more sensitive assay. Additionally, if we had allowed all of the patients in the entire cohort to receive a second dose of IFX, we presume that we would have seen a comparable rate of IIR as seen in most other studies. We believe that we averted IR in the 13 patients who were not allowed to continue with further doses. Two patients who had TDM prior to the second dose but were given IFX without the results at hand were found to have high ATI titers on retrospective review. Both were included in the non-TDM group and the serious IIR they experienced could have been prevented if the TDM protocol had been followed. Interestingly, in the 4 patients in the TDM group who had known ATI after the first dose but were allowed to continue with the therapy because the titers were deemed low, half were successfully treated long term. These data suggest that the level of ATI may allow for further stratification of patients.

**Table 3.** Infusion reactions with IFX restart in the literature

Author	No. of patients	Median drug holiday, months (*r*)	Total IR	IIR	DHR
Louis et al, 2012^24^	43	6.6 (IQR 4.0–10.8)	0 (during the first 3 doses)	0	0
Steenholdt et al, 2012^14^	108	28.7 (9.5–57.6)	17%	17%	Not reported
Farkas et al, 2013^23^	18	4 (IQR 3–8)	22%	22%	Not reported
Baert et al, 2014^18^	128	15 (6–125)	19.5%	11.7%	7.8%
Brandse et al, 2014^17^	29	14 (7–21)	10%	7%	3%
Molander et al, 2014^16^	14	Not reported	0	0	0
Gagniere et al, 2015^15^	61	37.6 (3.2–123.8)	39%	30%	7%

Abbreviations: DHR, delayed hypersensitivity reaction; IFX, infliximab; IIR, immediate infusion reaction; IQR, interquartile range; IR, infusion reaction.

In the non-TDM group, 5 patients developed IIR during the second dose, 4 of which were severe. Thus, although the absolute number of IIR with the first 2 infusions did not significantly differ between the TDM and non-TDM groups, qualitatively the IIRs were more severe in the non-TDM group. All severe IIRs occurred during the second restart dose, which is concordant with the findings by Steenholdt et al.^14^

DHR occurred in 12% of the cohort, 4 of 9 (44%) of them occurred in patients who were found to have positive ATI following the first restart dose and 3 of 9 (33%) in patients who had no ATI. In a meta-analysis by O’Meara et al^20^ assessing the risk of IR according to patients’ ATI status, there was not a difference in risk of DHR between ATI-positive and ATI-negative patients in 710 patients who experienced DHR. These findings suggest that patients should be warned, especially if the ATI assay is not drug-tolerant, that they may still develop a DHR, albeit the risk is low.

In this study, we were able to demonstrate that restarting IFX following a drug holiday is clinically effective, as over half of the patients who were restarted on IFX had medication persistence at 1 year. Younger age at restart of IFX and a shorter drug holiday were both associated with IFX persistence at 14 weeks and at 1 year. Additionally, we found that a longer drug holiday was associated with the presence of ATI. Concomitant use of an IMM at restart was only associated with short-term (14 weeks) IFX persistence. In agreement with our results, several studies have shown that IFX reintroduction is clinically effective and that patients with prior LOR to IFX can be recaptured with retreatment. Baert et al. studied a cohort of 128 patients (105 with CD, 23 with UC). They reported an 84.5% response at week 14, 70% at 1 year, and 61% at more than 4 years.^18^ Brandse et al^17^ included a cohort of 29 patients with CD who had been reinduced after sequential use of IFX and adalimumab. At the end of their 18-month follow-up period, 62% of the patients remained on IFX and 72% at 1 year. Another study published by Gagniere et al^15^ included 61 patients with CD who had also been reinduced with IFX after failing treatment with both IFX and adalimumab. The probabilities of remaining on IFX after reintroduction were 60% and 51% at 1 and 2 years, respectively. As in our study, short-term IFX persistence and long-term IFX persistence were associated with a shorter drug holiday. Furthermore, our study also showed that short-term IFX persistence and long-term IFX persistence were associated with a younger age at restart, which has not been previously described.

Concomitant use of IMM at restart has also been explored. It has been shown to have a protective role in reducing IR with IFX therapy^25, 26^ as well as improve the efficacy of IFX therapy,^2^ potentially due to the observed reduction in ATI formation with their use.^11, 27^

In our study, we did not find an association between IMM and ATI formation, IR or long-term IFX persistence, but we did find an association between the use of IMM and short-term IFX persistence (OR 3.5). However, as less than half of our cohort was started on IMM at the time of IFX restart, we likely did not have sufficient patients to reach firm conclusions about the effects of concomitant IMM use.

One of the limitations of this study is the small sample size. However, this is the first study that assesses proactive TDM in the setting of IFX reintroduction in real-life practice. Another limitation is the retrospective study design and lack of detailed historical data related to their first time on IFX. This also reflects that both our centers are tertiary referral centers wherein most patients have been on multiple therapies previously. Additionally, we used the persistence of IFX therapy as a surrogate marker of response, with the assumption that each treating physician is assessing their patients for response and not continuing therapy if the patient is not showing improvement on it. Having had disease activity scores pre-restart and during therapy as well as objective markers of biochemical response would have provided a better assessment of response to therapy. We did not include these in the study design as there was no standardization in how clinical assessments were performed by the different treating physicians, which is another limitation of this study. A prospective study, with a standardized assessment of disease activity after IFX restart, would be of value to better study this therapeutic option.

A strength of our study is provided by the different practices employed at each center which provides some range of approaches to this common clinical scenario. For example, we employed different premedication protocols and used different laboratories for TDM. Because different laboratories were used, we cannot set an ATI threshold above which patients should not be retreated. In general, the presence of ATI in the absence of detectable IFX should lead to IFX discontinuation, especially if measured only a few weeks after a dose. However, ATI threshold that should preclude continuation of therapy in the presence of detectable IFX is less clear. It has been demonstrated that drug-sensitive assays can underestimate ATI as they do not detect ATI in the presence of a drug. In our patient cohort, approximately half of the patients had TDM with drug-sensitive, rather than drug-tolerant, assays. As such, it is likely that additional patients in the TDM group had ATI that was not reported by the laboratory due to the type of assay, thus limiting the ability to flag patients who are at risk for IR or are less likely to benefit from retreatment with IFX. On the other hand, drug-tolerant assays have increased ATI detection which may incorrectly deter use of IFX, as it is known that low levels of transient, nonneutralizing ATI may be treated through with a low risk of IR and are clinically insignificant.^28^

## Conclusions

In summary, IFX can be safely and effectively restarted in patients with IBD who have had a drug holiday and who have failed multiple subsequent intervening therapies. A prospective study looking at markers of clinical response (disease activity scores and biochemical markers) and rates of remission is needed to study the specific efficiency of this treatment option. Based on our experience, we suggest that TDM be performed 1–3 weeks after the first IFX restart infusion as a means to identify patients at risk for IR at the second dose. Patients should be warned, especially if the ATI assay is not drug-tolerant, that they may still develop an IR and that they are at risk, albeit low (12%) of developing a DHR. It is a good idea to explain to patients typical signs and symptoms of a DHR including migrating polyarthralgia and jaw pain.^29^ The increased use of IMM in the non-TDM group may have also decreased IR in that group and in general should be considered in patients restarting IFX. This study challenges the previous dogma of “once exposed to infliximab, if you stop, you cannot go back.” Given the need for additional therapies and options for patients with IBD, reintroduction of IFX should be considered in appropriate patients.

## Data Availability

The data that support the findings of this study are available on request from the corresponding author.
